# Immune landscape and prognostic index for pancreatic cancer based on TCGA database and in vivo validation

**DOI:** 10.1186/s12885-023-10597-9

**Published:** 2023-02-10

**Authors:** Pan-ling Xu, Chien-shan Cheng, Ting Wang, Shu Dong, Ping Li

**Affiliations:** 1 Department of Chinese Integrative Medicine Oncology, The First Affiliated Hospital of Anhui Medical University, 230022 Hefei, Anhui China; 2 Department of Integrative Oncology, Fudan University Shanghai Cancer Center, 200032 Shanghai, China; 3 Department of Oncology, Shanghai Medical College, Fudan University, 200032 Shanghai, China

**Keywords:** Pancreatic cancer, Immune landscape, Prognosis, M0 macrophage

## Abstract

The immunotherapy efficacy on pancreatic cancer remains unsatisfactory. Therefore, it is still necessary to further clarify the pancreatic immune cell infiltration and search for immune-related prognostic indicators. We analyzed the 135 pancreatic cancer patients’ data retrieved from the TCGA database for the immune cell infiltration, tumor microenvironment score and the correlation of the immune cells, followed by identification of prognostic immune clusters and genes clusters. The R language was used for the immune score calculation, and immune cells proportion related survival differences identification. The function of immune cells was verified through datasets in the GEO database and in vivo experiments. The results showed that M0 Macrophages had negative relations to CD8 + T cells and immune scores. There were differences in median survival in ICI clusters, gene clusters, and immune score groups (*p* < 0.05). M0 macrophages accounted for more than 9.8%, indicating a poor prognosis, while T cells accounted for more than 9.2%, indicating a good prognosis. In vivo results showed that M0 macrophages promote pancreatic cancer growth. Elimination of M0 macrophages may be a hopeful strategy against pancreatic cancer.

**Supplementary Information:**

The online version contains supplementary material available at 10.1186/s12885-023-10597-9.

## Highlights

M0 macrophages accounted for more than 9.8%, indicating a poor prognosis in pancreatic cancer.

M0 macrophages proportion were negatively correlated with CD8 + T cells, and immune scores.

M0 macrophages promote pancreatic cancer growth in vivo.

Elimination of M0 macrophages may be a promising strategy against PAAD.

**Supplementary Information:**

The online version contains supplementary material available at 10.1186/s12885-023-10597-9.

## Introduction

As a particularly deadly and aggressive cancer, pancreatic adenocarcinoma (PAAD) is the fourth common cause of cancer-related mortality in the United States [1]. Despite advances in diagnostic methods, perioperative management, chemotherapy, and other systematic treatments, the improvement in PAAD prognosis remains limited over the past decade. Immunotherapy has shown promising therapeutic outcomes in various tumors [2–5], but in pancreatic cancer, immunotherapy has not shown the desired effect. Currently, traditional clinicopathological parameters novel tumor and molecular biomarkers have been used in the prognostic evaluation and patient subgroup identification in PAAD patients. To further improve the management of PAAD and identify a patient subgroup that can benefit from immunotherapy [6], the research on immune-related potential predictors and therapeutic targets is critical.

The crucial reason leading to drug resistance and poor treatment response of PAAD in the complex tumor microenvironment (TME) is characterized by the increased extracellular matrix deposition and rare immune cell infiltration [7, 8]. The stromal components of the TME comprise fibroblasts, immune cells, blood vessels, bone marrow cells, and extracellular matrix. The TME stroma has been proved to correlate with treatment resistance and immunosuppressive effects that contribute to immunotherapy tolerance [9]. Although several clinical trials attempted on T cell checkpoint inhibitor in PAAD, minimal therapeutic benefits were obtained, stressing the urgent need to deepen the understating of the immune microenvironment in PAAD. The investigation on immune cell subtype and their prognostic significance, along with their role in tumor progression, can aid in identifying appropriate biomarkers and potential therapeutic targets for future treatment of PAAD.

The purpose of this study is to find out the immunological traits and immunoprognostic markers of PAAD and provide a perspective for intervention. Through the integration of the pancreatic cancer ribonucleic acid (RNA) sequencing data, the Cancer Genome Map (TCGA) (https://portal.gdc.cancer.gov/) and the Gene Expression Omnibus (GEO) [10] (https://www.ncbi.nlm.nih.gov/geo/) databases were mined to compare cancer features in many dimensions. We screened for prognostic immune indicators from different dimensions through bioinformatics and verified them through datasets in the GEO database and in vivo experiments. The present study results aim to provide an auxiliary basis for predicting pancreatic cancer outcomes and identifying patient subgroups for immunotherapy in the future.

## Materials and methods

### TCGA and GEO data collection and preprocessing

The RNA sequencing data and clinical features on PAAD were searched out from the TCGA database. The R software 4.0.3 [11] and the Practical Extraction and Report Language (Perl) (https://www.perl.org/about.html) [12] were used for the RNA matrix data translation. Patients with incomplete clinical data were excluded, and 135 patients’ data were eventually included.

The keywords “pancreatic/pancreas,” “tumor/cancer,” and “survival” were used to search and download the data from the GEO database for PAAD. The inclusion criteria were met by 336 data sets in total. The following criteria were used to further filter the results: (1) human pancreatic ductal adenocarcinoma tissue; (2) ≥ 30 samples; (3) transcriptome expression data; (4) The data included patients’ survival data. In the end, three data sets satisfied the aforementioned requirements. To assess the quality of the included data sets, the “affyPLM” program in R was used. Then background normalization and correction were performed using robust multi-chip analysis (RMA).

### Immune cell infiltration and TME scores

By calculating the composition of immune cells in TME, the enrichment of immune infiltrating cells was finally determined. And the proportion of immune cells was computed using CIBERSORT (https://cibersortx.stanford.edu/) code, a human leukocyte subtype expression matrix deconvolution tool based on linear support vector regression [13], in the R program for further study. The “estimateScore” package in R were severed for TME scores calculation.

### Immune cells correlation analysis

To determine the correlation among differential immune cells, we conducted a correlation test between the content of immune cells by using the immune cell infiltration and TME scores. A *p-*value of less than 0.05 indicates the presence of a correlation between immune cells.

### Immune clusters and survival analysis

Using the R package “ConsensusClusterPlus” for class discovery, unsupervised clustering was used to compare the makeup of the immune cells. K is the quantity of clusters produced by unsupervised clustering [14]. The ideal K was evaluated using the consensus heatmap and cumulative distribution function (CDF). To determine whether there is a survival difference between immune clusters, the R packages “survival” and “survminer” were employed. A p-value of less than 0.05 indicates a significant difference in survival. Using the “ggplot2” and “ggpubr” packages, boxplots were created to display the immunological clusters’ immune cell makeup and gene expression.

### Differentially expressed genes(DEGs) clusters and survival analysis

With the |logFC| filter > 0.585 and adj.P.Val. filter 0.05, the DEGs in various immune clusters were found using the R package “Limma”. The package “ConsensusClusterPlus” was used again for class discovery to get the gene clusters. The ideal K was evaluated using the CDF and consensus heatmap. The “survival” and “survminer” R packages were then used to find a survival differential between gene clusters. A difference in survival between gene clusters can be detected by a p-value of less than 0.05. Using the “ggplot2” and “ggpubr” packages, a plot of the gene expression in several gene clusters and the makeup of immune cells was created.

### Immune score clusters and survival analysis

Each sample’s immunological score was determined using R’s principal component analysis (PCA). Through an orthogonal transformation, the statistical technique PCA transforms correlated data into variables that are not linearly associated. Principal components [15] refer to this collection of variables following transformation. The ideal cutoff value was established based on the tumor immune score and patient survival time. Based on the optimal cutoff value, patients were divided into high and low groups, and the disparity in survival rates between the two groups was investigated. A p-value of 0.05 demonstrates the difference in survival rates between the high and low groups.

### Immune cells proportion and survival analysis

The immune cells proportion had been calculated using the CIBERSORT code in the R program. Combined with the survival data, the X-tile software 3.6.1 was used to obtain the optimal cutoff value [16]. Patients were split into groups with high and low immune cell proportions, and survival differences between the two groups were examined. A significant difference in survival between the high and low groups is shown by a p-value of less than 0.05.

### Acquisition of fluorescent cancer cells

Pancreatic cancer cell line Panc-02 was purchased from Frederick National Laboratory for Cancer Research (Frederick, MD, USA) and cultured in Dulbecco’s modified Eagle’s medium (Gibco, USA) with supplements of 10% fetal bovine serum (Gibco, USA), 100 µg/ml penicillin, and 100 µg/ml streptomycin (Gibco, USA) at 37 °C in an atmosphere with 5% CO_2_. Shanghai Genechem Co., Ltd. supplied the negative control lentivirus (U6-MCS-Ubiquitin-EGFP-IRES-puromycin) with a concentration of 1E + 9 TU/ml (Shanghai, China). Using 2 mL of media, Panc-02 cell suspensions (2 × 10^5^ cells/mL) were sown on 6-well plates and preincubated overnight. Following adhesion, the cells were cultured for 48 h in 2 mL of media containing lentivirus 1 × 10^7^ TU/mL and HitransG A infection enhancer (25 ×) 80 µL. After that, the cells were grown for 48 h in a new medium. The cells were then grown in a brand-new screening medium that contained puromycin (Beyotime, Shanghai, China) at a concentration of 5 µg/mL. After twenty days of growth, a Panc-02 cell line expressing EGFP fluorescence was produced. Puromycin was added to a fresh medium containing Panc02 cells at a concentration of 2.5 µg/mL, and the purity of the cells was checked by flow cytometry (CytoFLEXS, Beckman COULTER, USA).

### Acquisition of tumor single-cell suspension

Male, 5–6 week C57BL/6J mice were purchased from Shanghai Jihui Laboratory Animal Care Co., Ltd. (Shanghai, China) and kept in laminar flow cabinets with food and water in a pathogen-free environment. The Fudan University Shanghai Cancer Center’s committee on the ethics of animal research gave its approval to this study protocol (No. FUSCC-IACUC-2,021,540). After one week of adaptive feeding, the mice were subcutaneously inoculated with 2 × 10^6^ fluorescent cancer cells on the right posterior back. The mice were put to death by intraperitoneal injection of 250 mg/kg pentobarbital sodium when the subcutaneous tumors had grown to a diameter of roughly 10 mm. The subcutaneous tumors were then removed. As previously reported, the preparation of a single-cell suspension of the subcutaneous tumors was performed with slight modifications [17]. In brief, subcutaneous tumors were digested into single-cell suspensions using gentleMACSTM Octo Dissociator (Miltenyi Biotec, Germany) with enzymolysis solution formulated with 0.5% Bovine Serum Albumin (BSA) (Sigma-Aldrich, Shanghai, China), collagenase I 10,000 U/mL (Sigma-Aldrich, Shanghai, China), Dispase II 32 mg/mL (Roche, Basel, Switzerland), and DNAase I 5 MU/mL (Calbiochem, Germany) [18]. A 70 μm cell strainer was used to separate the tissue suspension from the tumor after 20 min of enzymatic dissociation to produce a single-cell suspension.

### The ratio of Panc02 cells to M0 macrophages in the tumor

After being blocked with CD16/CD32 (Cat. 553,141, BD Pharmingen™, USA), single-cell suspension (1 × 10^6^ cells) was incubated with a flow panel for 30 min avoiding light, the panel included APC-CY7 conjugated CD45 (Cat. 557,659, BD PharmingenTM, USA), PE-conjugated F4/80 (Cat. 565,410, BD PharmingenTM, USA), APC-R700 conjugated CD86 (Cat. 565,479, BD PharmingenTM, USA), and AF-647 conjugated CD206 (Cat. 565,250, BD PharmingenTM, USA). The ratio of Panc02 cells to M0 macrophages was then calculated using flow cytometry (CytoFLEXS, Beckman COULTER, USA) to identify the single-cell suspension.

### Acquisition of M0 macrophages

Per 10^7^ tumor cells, 10 µL of Anti-F4/80 MicroBeads UltraPure (Miltenyi Biotec, Germany) were added, and the mixture was then incubated darkly in the refrigerator for 15 min. The F4/80 + macrophage cells were isolated from tumor single-cell suspension using MACS Separator (Miltenyi Biotec, Germany) and detected by flow cytometry (CytoFLEXS, Beckman COULTER, USA) to assess for purity. F4/80 + macrophages were incubated with CD86 and CD206 antibody for 30 min avoiding light, then a flow sorter (MoFlo Astrios EQ, Beckman COULTER, USA) was used to sort out F4/80 + CD86-CD206- cells.

### ***In vivo*** studies

Twelve male C57BL/6J mice, 5–6 weeks old, were housed in the Fudan University Shanghai Cancer Center’s laboratory animal facility. The mice were split into two groups of six each after a week of adaptive feeding. Panc02 2 × 10^6^ cells were subcutaneously injected into one group, Panc02 1.34 × 10^6^ cells and M0 macrophages 0.66 × 10^6^ cells were subcutaneously injected into the other group. Every three days for seven days following inoculation, the body weight and tumor diameter were assessed. The mice were euthanized 24 days after implantation with a 250 mg/kg intraperitoneal injection of pentobarbital sodium, and the subcutaneous tumor was removed, dissected, and weighed. For further processing, the tumors were fixed in a 4% paraformaldehyde solution (Beyotime, Shanghai, China).

### Immunohistochemistry and immunofluorescent

The paraffin-embedded fixed tumors were cut into 4 μm sections. Using the Ki-67 antibody, immunohistochemistry (IHC) staining was carried out on the slides (1:2000, Cat. AB15580, Abcam, UK). The slides were treated with diluted secondary antibodies for one hour at room temperature after spending the night in humidifying chambers set at 4 °C. The slides were then loaded, dehydrated, and dyed using DAB substrate kits (Carrier Laboratory, Burlingame, CA, USA) for one minute, before being examined using a light microscope (200×, Leica, Wetzlar, Germany).

Slides were incubated with CY3 conjugated F4/80 antibody (1:200, 70,076, CST, Boston, USA), FITC conjugated CD86 antibody (1:300, 19,589, CST, Boston, USA), and CY7 conjugated CD163 antibody (1:500, ab182422, Abcam, UK). After an overnight incubation at 4 °C in a humid environment, the antibodies fluorescent mounting material (Dako, Denmark) was used to mount stained slides so they could be seen under a confocal microscope (Carl Zeiss LSM780, Germany).

### Statistical analysis

Unless otherwise stated, every experiment was carried out in triplicate. The mean and standard deviation are used to present all the data. Using SPSS software 23.0, the Student’s t-test was used to evaluate the data (SPSS Inc., Chicago, IL, USA). Statistical significance was determined by a p-value of less than 0.05.

## Result

### The Immune Cell Infiltration Landscape in PDAC

**Fig. 1:**
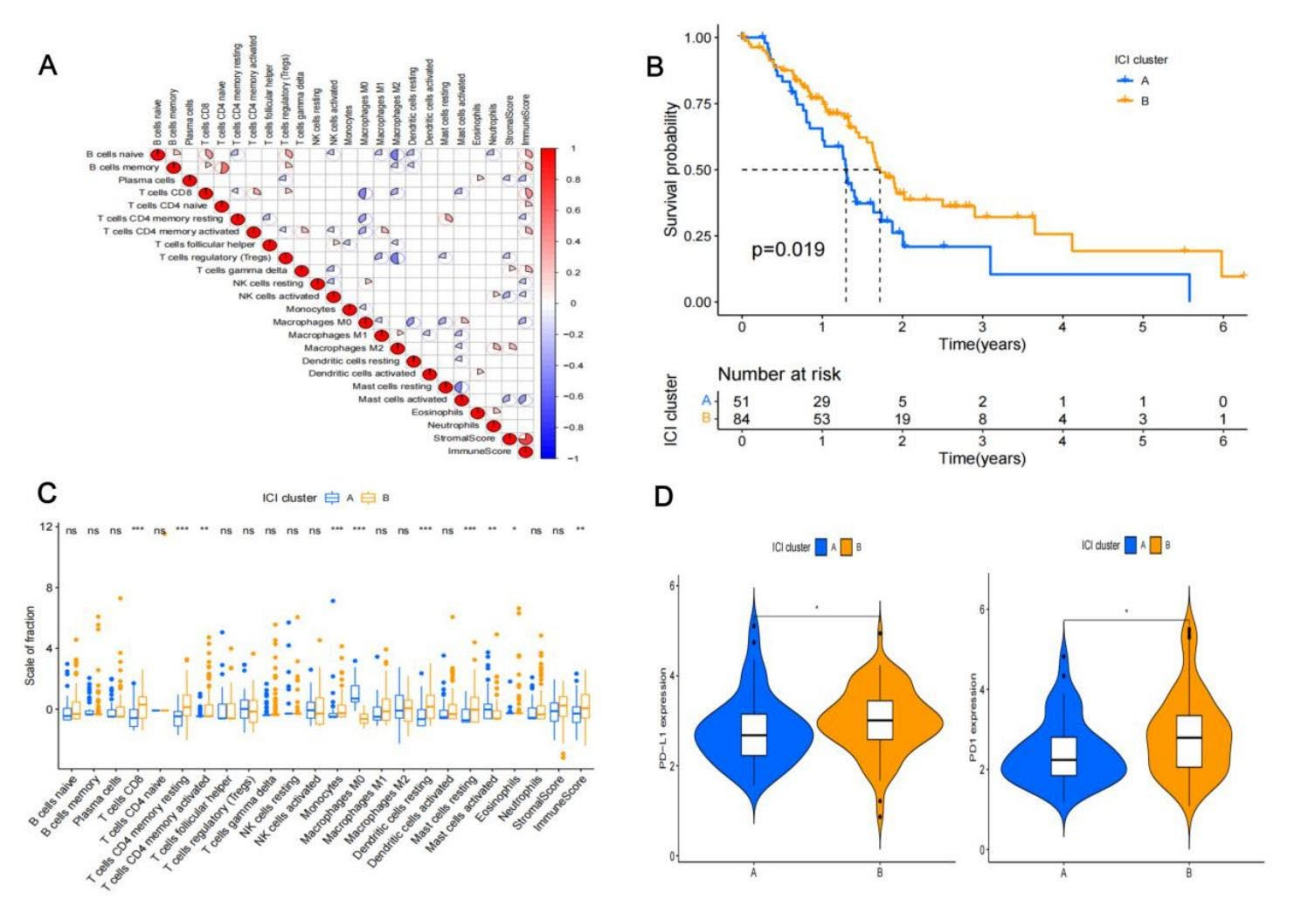
The immune cells infiltration and ICI clusters function in pancreatic cancer. (A) The correlation between immune cells in pancreatic cancer tissue. Red means positive correlation, blue means negative correlation (larger the sector area, smaller the *p* value); (B) Two ICI clusters with prognosis significance; (C) Immune cells proportion in two ICI clusters (ns: no significance; *: *p* < 0.05; **: *p* < 0.01; ***: *p* < 0.001 ); (D) PD1 and PD-L1 expression in two ICI clusters

A total of 135 PAAD patients’ clinical information and transcription were included in this study. Tumor microenvironment scores and immune cell enrichment levels in PAAD tissues were evaluated using the CIBERSORT and ESTIMATE algorithms. Figure 1A illustrates the analysis of the correlation between immune cells in the tumor microenvironment of PAAD. A negative correlation existed between M0 macrophages and immunological scores, CD8 + T cells, and activated dendritic cells. The relationship between CD8 + T cells and immunological score was favorable.

The “ConesusClusterPlus R” program was used to find two distinct ICI clusters. The median survival in the two groups was different (ICI cluster A and ICI cluster B, 432 vs. 557 days, respectively; *p* = 0.019) (Fig. 1B). We analyzed the immune cell composition in PAAD to further clarify the underlying biological variations that result in various ICI clusters (Fig. 1C). With high levels of M0 macrophages and active mast cells, ICI cluster A was associated with a poor prognosis; in contrast, ICI cluster B was associated with higher levels of CD8 + T cells, CD4 + memory T cells, monocytes. Each ICI subtype’s immune checkpoint PD1 and PD-L1 expression levels were assessed. Compared to the ICI cluster A subtype, the ICI cluster B subtype displayed greater expression of PD1 and PD-L1 (Fig. 1D).

### Immune Gene clusters with prognosis

**Fig. 2:**
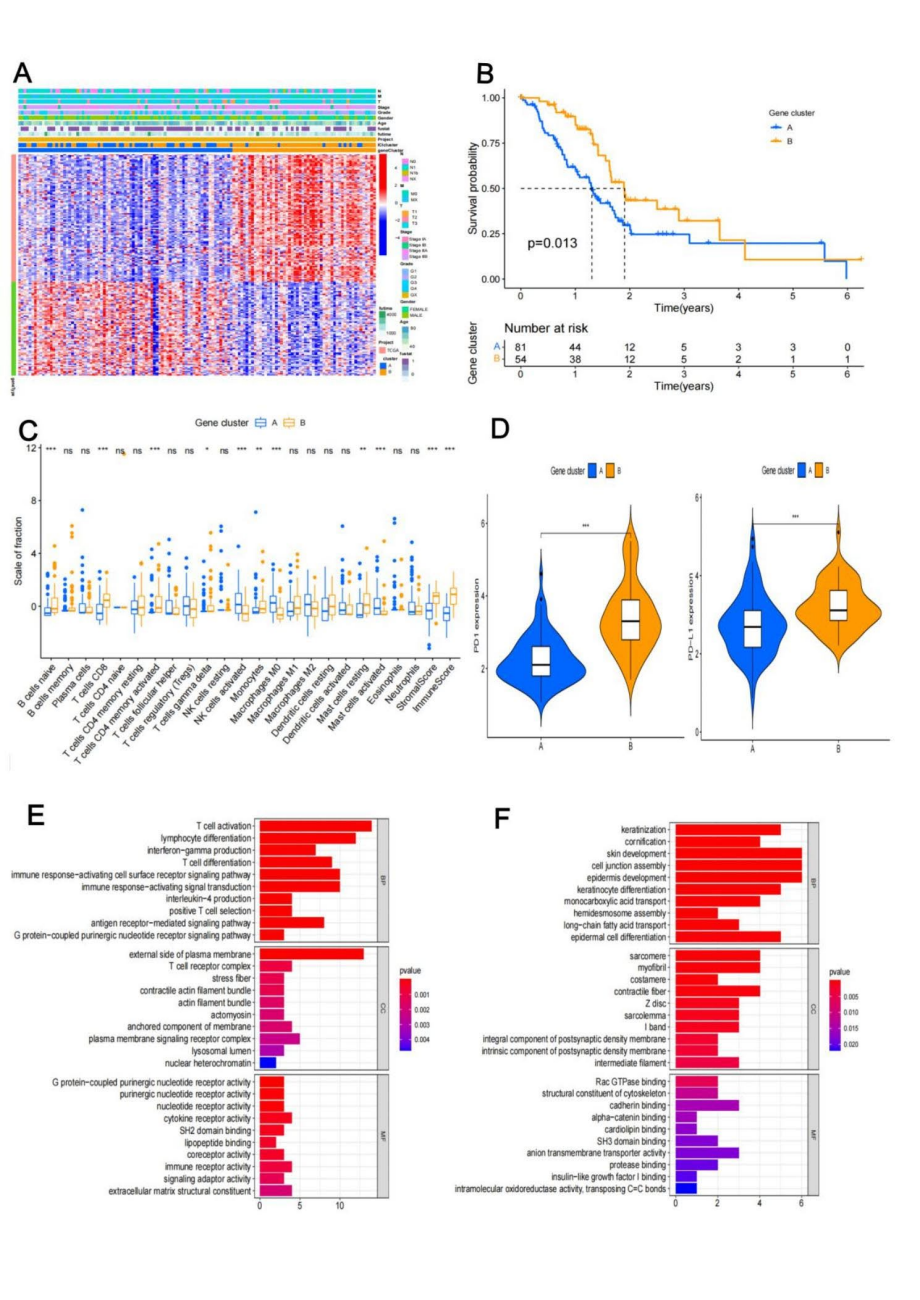
Immune gene clusters function and prognosis. (A) The heat map about 184 DEGs transcriptome profiles; (B) Two gene clusters with prognosis significance; (C) immune cells proportion in two gene clusters (ns: no significance; *: *p* < 0.05; **: *p* < 0.01; ***: *p* < 0.001 ); (D) PD1 and PD-L1 expression in two gene clusters. E and F. GO functional enrichment analysis, T cell activation and lymphocyte differentiation play an important role in immune response

To find DEGs between the ICI subtypes mentioned above, we used the “limma” package in the R software. There were 184 DEGs between ICI clusters. Two immunological gene clusters were found using an unsupervised clustering methodology. The ICI gene signature A refers to the 106 gene signatures that have a positive correlation with the gene cluster, while the ICI gene signature B refers to the remaining 106 gene signatures. The heatmap displayed the transcriptome profiles of 184 DEGs discovered by various gene groupings (Fig. 2A). Patients in gene cluster B had a better prognosis than those in gene cluster A, according to a Kaplan-Meier analysis (median OS: 468 vs. 571 days; *p* = 0.013) (Fig. 2B).

In gene cluster A, there were significant numbers of mast cells, M0 macrophages, and activated NK cells; in gene cluster B, there were significant numbers of naive B cells, CD8 + T cells, activated CD4 + memory T cells, gamma delta T cells, monocytes, and resting mast cells (Fig. 2C). The gene cluster B subtype showed higher expression of PD1 and PD-L1 when compared to the gene cluster A subtype (Fig. 2D). The cell junction assembly, keratinization, and cornification were the main areas where the A signature genes were concentrated, according to the GO functional enrichment study. While the primary areas of focus for B signature genes were T cell activation, lymphocyte differentiation, and the activating cell surface receptor signaling pathway of the immune response (Fig. 2E and F).

### The Immune score construction with prognosis

**Fig. 3:**
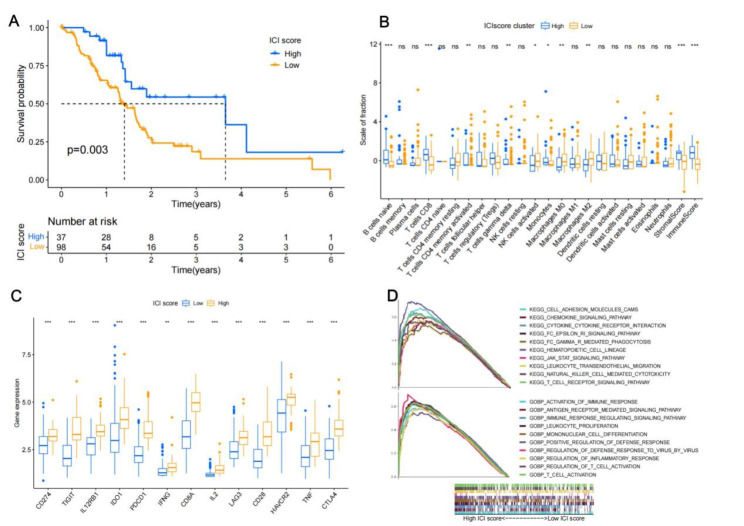
The immune score construction with prognosis. (A) Immune score groups with prognosis significance; (B) immune cells proportion in immune score groups; (C) The expression levels of T-cell activation and tolerance genes in two immune score groups; (D) Gene set enrichment analysis

We employed PCA to determine the immunological score in PAAD patients. Using the best cutoff values acquired from the X-tile program, patients were split into two groups with high or low immunological ratings. A score of more than 3.3 indicates a high immune score, while a score of less than 3.3 indicates a poor immune score. Survival curves were plotted using the R software’s “survival” and “survminer” package (Fig. 3A). High immunological ratings in PDAC patients were associated with a better prognosis (median OS: 1351 vs. 474 days; p = 0.003). High levels of naive B cells, CD8 + T cells, activated CD4 + memory T cells, gamma delta T cells, monocytes, and resting mast cells were present in the group with a high immunological score. High numbers of activated NK cells, M0 macrophages, and M2 macrophages were found in the group with a poor immunological score (Fig. 3B). Then, we contrasted the T-cell activity and tolerance indices between the groups with high and low scores. The findings demonstrated that the high-score group had significantly higher expression levels of T-cell activation and tolerance genes than did the low-score group (Fig. 3C). The high immunological score group had the highest concentration of cytokine pathways and immune system activation, according to the gene set enrichment analysis (GSEA) (Fig. 3D). According to this finding, PAAD patients with high immunological ratings had a better prognosis.

### The roles of M0 macrophages and CD8 + T cells in Prognosis Prediction

**Fig. 4:**
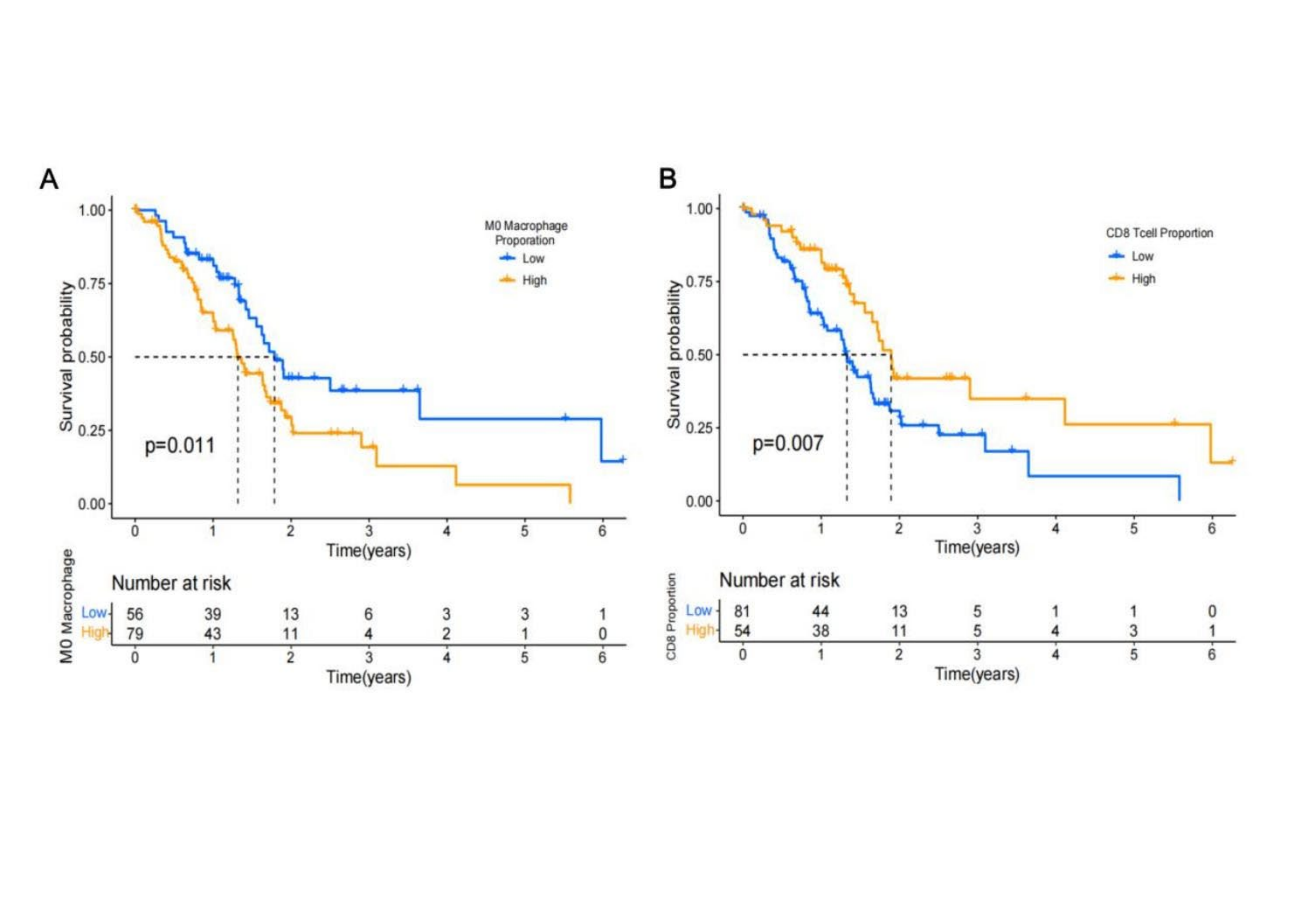
M0 macrophages and CD8 + T cells with prognosis significance. (A) M0 macrophages high group means a poor prognosis; (B) T cells high group means a good prognosis

We associated the proportion of immune cells in a patient’s tumor with overall survival (OS) and used X-tile to determine the optimal cutoff value. The results showed that M0 macrophages accounted for more than 9.8% of the immune cells, indicating a poor prognosis. In comparison, T cells accounted for more than 9.2% of the immune cells, indicating a good prognosis (Fig. 4A and B).

**Fig. 5:**
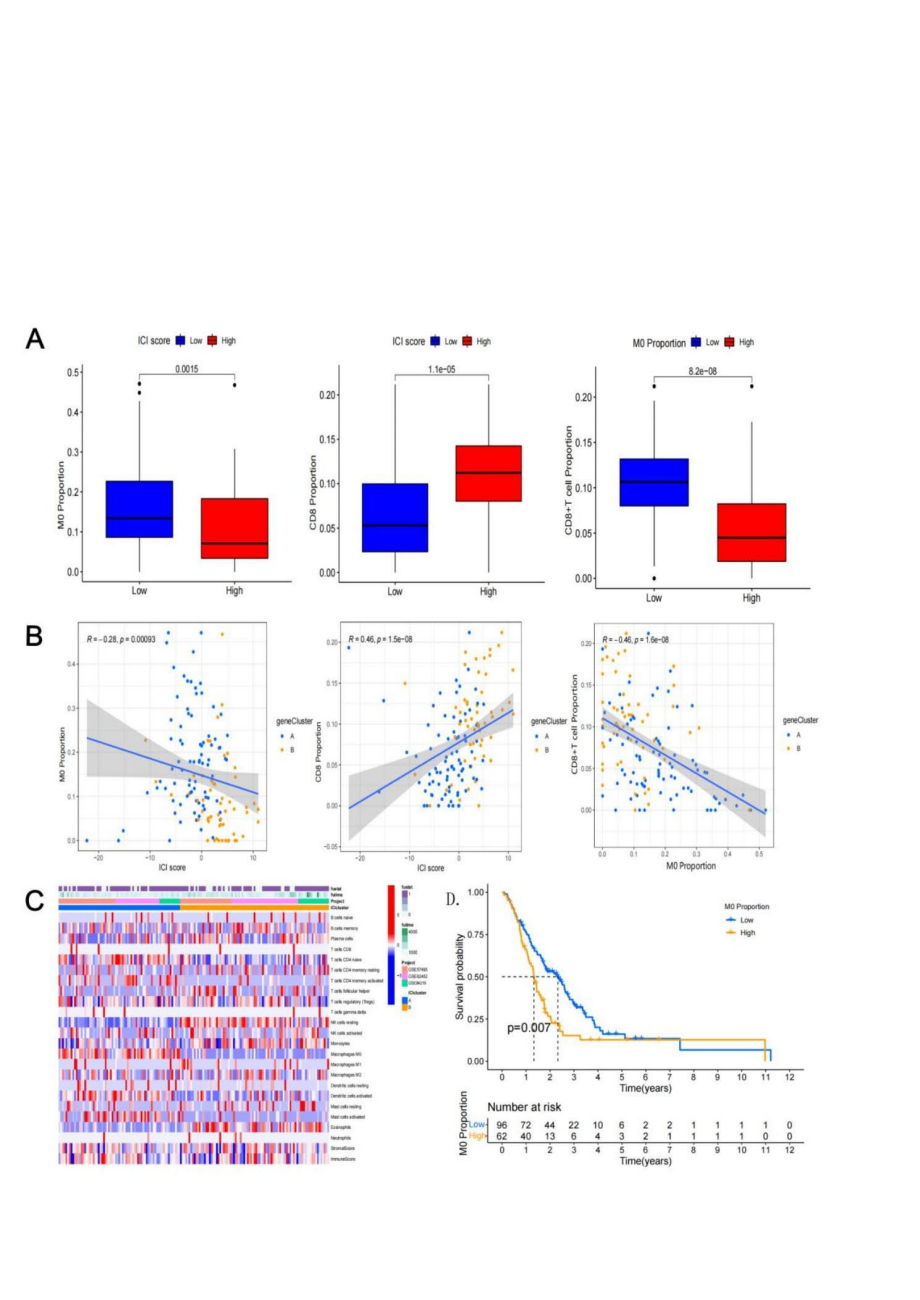
The M0 macrophages’ role in PAAD. A and B. The relationship among M0 macrophages, CD8 + T cells, and immune score; C. The heat map in GEO data sets; D. M0 macrophages high means a poor prognosis in GEO data sets

The proportion of M0 macrophages was negatively correlated with the immune score, while that of CD8 + T cells was positively correlated with the immune score (Fig. 5A and B). To clarify the effect of M0 macrophages, we screened out three datasets (GSE57495; GSE62452; GSE84219) from the GEO database and analyzed the infiltration of the immune cells (Fig. 5C). The results showed that PAAD patients with a high intra-tumoral proportion of M0 macrophages had a poor prognosis (Fig. 5D).

### M0 macrophages promote pancreatic Cancer growth in vivo

**Fig. 6:**
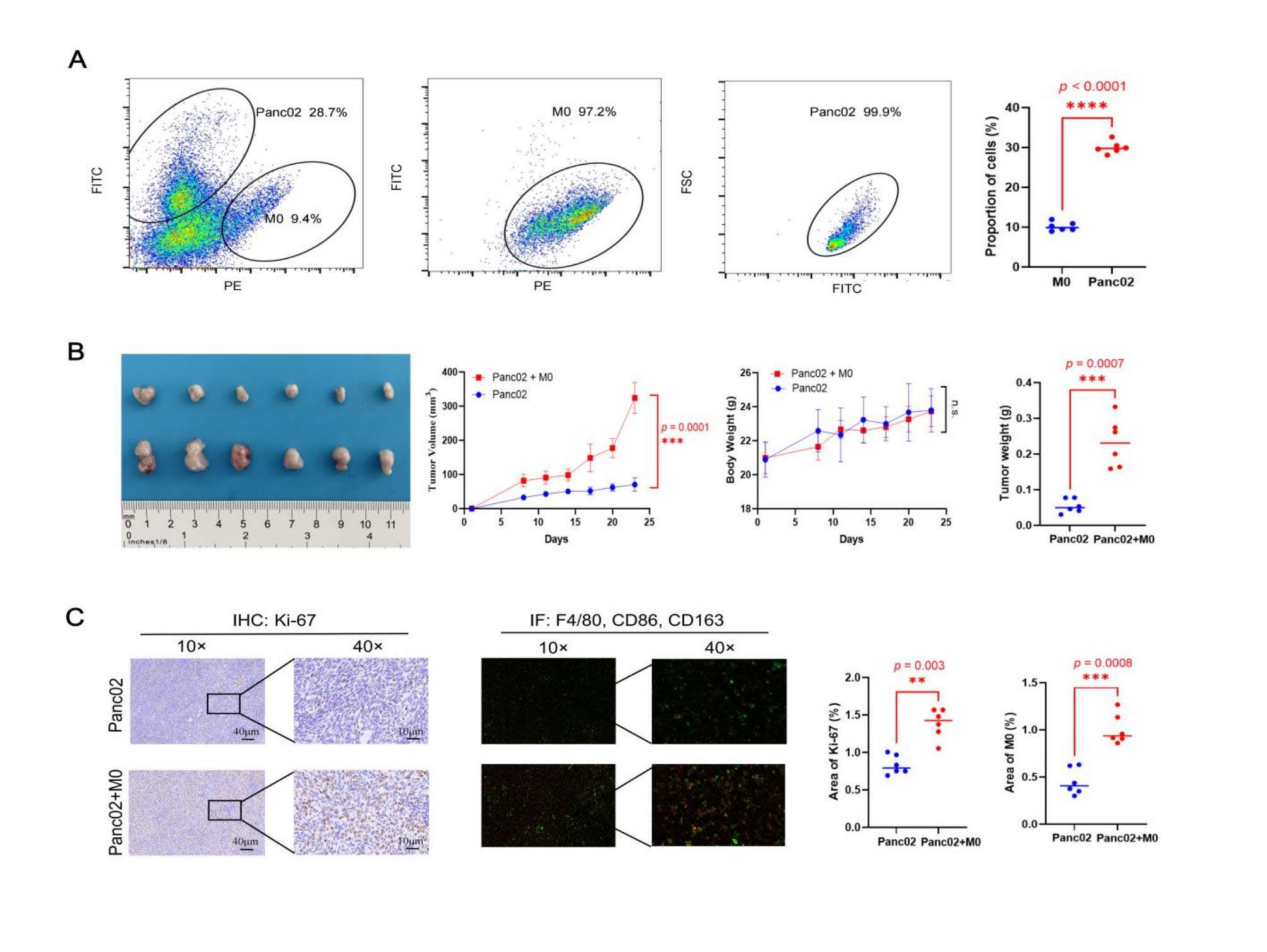
M0 macrophages promoted pancreatic cancer growth. (A) the ratio of Panc02 cells to M0 macrophages and the purity of cells; (B) The tumor volume and tumor weight in two groups; (C) The expression of Ki-67 and the proportion of M0 macrophages in two groups

In order to confirm the impact of M0 macrophages on pancreatic cancer, a mice subcutaneous xenograft was created. The findings revealed a roughly 3:1 ratio between Panc02 cells and M0 macrophages. More than 97% of the cells were fluorescently expressed Panc02 cells and separated M0 macrophages (Fig. 6A). Tumor volume and weight significantly increased in the M0 macrophages plus Panc02 group after 24 days following tumor inoculation (p<0.05) (Fig. 6B). Results of immunohistochemistry and immunofluorescent analysis showed that the M0 macrophages mixed with the Panc02 group significantly increased the expression of Ki-67 and the fraction of M0 macrophages (Fig. 6C). These results suggested that pancreatic cancer growth in vivo is stimulated by M0 macrophage. And the percentage of CD8 + T cells between the two groups was shown in Supplementary Fig. 1. The results showed that the percentage of CD8 + T cells was higher in the group without M0 macrophages.

## Discussion

Over the past decade, immunotherapy has transformed the paradigm of cancer treatment [19]. However, PAAD patients showed an abysmal response towards immunotherapy, and therefore known as “cold” tumors, characterized by abundant non-cancer components, such as immune cells, fibroblasts, and stroma in the tumor microenvironment [20]. The results in our present study, consistent with previous reports [21], showed that cancer cells accounted for approximately 30% of the cell count in a murine subcutaneous tumor xenograft. To our surprise, the results of this study revealed that the number of CD8 + T cells in the immunological microenvironment was noticeably low; a recent study contends that a subpopulation of CD8 + T cells that express CD25 and FOXP3 can limit the activity of cytotoxic CD8 + T cells [22–24]. As suggested by the existing immunotherapy clinical trials [25, 26], the poor response of current T-cell-based immunotherapies may be due to insufficient activation of endogenous T cells. So, it is very important to further examine the immune microenvironment of pancreatic cancer and explore new therapeutic strategies.

With the development of bioinformatics and the free sharing of databases, we tried to find the characteristics and prognosis of pancreatic cancer from different dimensions. Our results suggested a significant prognostic role of ICI and gene clusters, and unsupervised clustering can only cluster into a specified number of classes, but it can not explain what each class represents. Therefore, quantitative evaluation immune score from the ICI and gene clusters was analyzed, suggesting superior prognostic predictability. This positive correlation of immune score and prognosis is consistent with previous reports [8]. We discovered that the quantity and functionality of CD8 + T cells may be connected to the immunological score level. The subgroup analysis with a favourable prognosis revealed a higher percentage of CD8 + T cells, and function enrichment showed the existence of immune cells that are activated. Together, our results not only demonstrate that the immune score is a reliable prognostic biomarker in PAAD but also provide a hint for the exogenous administration of activated T cells, such as the recent reports of the effectiveness of chimeric antigen receptor T-cell (CAR-T) therapy in murine patient-derived tumor [27–29].

In tumor tissues, M0 macrophages, sometimes called as naive macrophages, can polarize into M1 or M2 macrophages to act anti-tumor or promoting-tumor function. According to our subgroup analysis, which was consistent with earlier results [30], The more M0 macrophages in the tumor tissue, the lower the survival of PAAD patients. The proportion of activated dendritic cells, CD8 + T cells, and immunological scores were also found to be negatively correlated with M0 macrophages. It is important to note that several research [31] concentrated on the phenotypes and activities of M1 and M2 macrophages, especially the involvement of M2 macrophages in the promotion of tumors. However, the role of M0 macrophage in PAAD progression received limited attention [32]. Currently, M0 macrophages lack specific surface markers, although CD86 + is widely used to mark M1 macrophages and CD206 + is used to mark M2 macrophages, respectively [33, 34]. In this study, we employed F4/80 microbeads as pan-macrophages markers, followed by the subsequent removal of CD86 + or CD206 + cells to sort and purify M0 macrophages. Our in vivo experiment showed that M0 macrophages could promote the growth of pancreatic cancer. Yet, whether M0 promotes tumor growth through direct contact with tumor cells or by inhibiting T cell function remains unclear. The role of activated dendritic cells also warrants further investigation.

The study had a number of restrictions. To start, 135 patients’ M0 data from the TCGA database were chosen for investigation in this study, and 158 patients’ data from the GEO database were used for verification. The study’s sample size can be increased further. Second, results on the percentage of CD8 + T cells in the TCGA database were likewise favorable. However, the GEO database lacked the necessary transcriptome information, so it was unable to confirm the function of CD8 + T cells in GEO database. So, we did not further investigate the function of CD8 + T cells in subsequent animal experiments. Third, there are no clear surface markers for the phenotype of M0 macrophages, and we obtained M0 macrophages by step-wise gating elimination method. Therefore, further investigation of M0 macrophages function and treatment on M0 macrophage will be an exciting direction.

In this study, data mining and bioinformatics methods were employed to study the immune landscape of pancreatic cancer. The results indicated that immune score and M0 proportion are good predictors of prognosis. Animal experiments showed that M0 macrophages promoted pancreatic cancer growth. Together, the present study provides a prognostic prediction model and suggests that targeting M0 macrophages may be a novel strategy for future PAAD treatment.

## Electronic supplementary material

Below is the link to the electronic supplementary material.

Supplementary Material 1

## Data Availability

These data were derived from the following resources available in the public domain: [TCGA database; https://portal.gdc.cancer.gov/].
