# Supplementary material for: Immune landscape and prognostic index for pancreatic cancer based on TCGA database and in vivo validation
Source: BMC Cancer. 2023 Feb 10;23:139. doi: 10.1186/s12885-023-10597-9 (PMC9912589; doi:10.1186/s12885-023-10597-9)
Supplement: Supplementary file 1 — Supplementary Material 1 [file 12885_2023_10597_MOESM1_ESM.docx]

[Supplementary F](javascript:;)igure 1


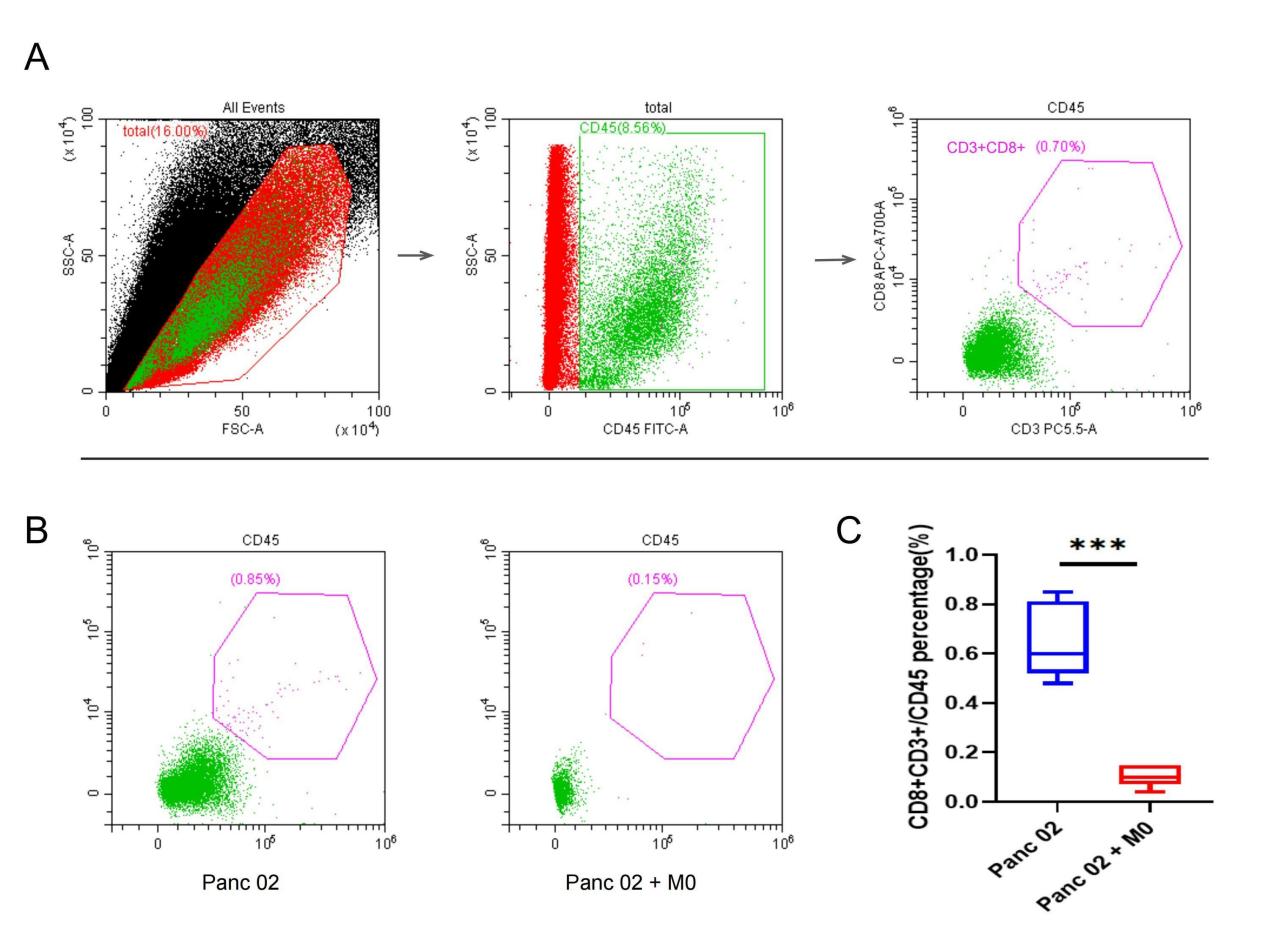


Supplementary Figure 1. CD8+T cell percentage between two groups. A. Flow cytomegate strategy; B. CD8+T cell percentage in Panc 02 and Panc02+ M0 groups; C. The *p* value between two groups (***: *p* < 0.001).
